# Richter Transformation of Chronic Lymphocytic Leukemia: A Review of Fluorodeoxyglucose Positron Emission Tomography–Computed Tomography and Molecular Diagnostics

**DOI:** 10.7759/cureus.968

**Published:** 2017-01-09

**Authors:** Faiq Shaikh, Amna Janjua, Frederick Van Gestel, Adeel Ahmad

**Affiliations:** 1 Imaging Informatics, University of Pittsburgh Medical Center, Pittsburgh, PA.; 2 Medicine, Army Medical College, Rawalpindi, Pakistan; 3 3rd Master Medicine, Catholic University of Leuven; 4 Dermatopathology/Dermatology/Pathology, Private Practice, Beckley, WV.

**Keywords:** richter, transformation, cll, pet, molecular, fdg

## Abstract

Chronic lymphocytic leukemia (CLL) is a low-grade B-cell proliferative disease with a generally indolent course. In a few cases, it undergoes transformation and becomes a more aggressive malignancy, such as diffuse large B-cell lymphoma (DLBCL). This process, which is called Richter transformation (RT), is often detected too late and is associated with a poor prognosis. There are multiple molecular diagnostic approaches to detect RT in preexisting CLL. Metabolic imaging using 18-fluorine fluorodeoxyglucose positron emission tomography–computed tomography (18F-FDG PET/CT) can be a very useful tool for early detection of RT and which can hence allow for timely intervention, thereby improving the patient’s chances of survival.

## Introduction and background

Chronic lymphocytic leukemia (CLL) is the most commonly diagnosed adult leukemia in the United States and Canada [1]. It is characterized by the proliferation and accumulation of phenotypically distinct monoclonal B-cell lymphocytes derived from blood, marrow, or lymph nodes. It is generally an indolent, low-grade lymphoproliferative disorder; however, in about 2%–10% of cases, patients develop a more aggressive disease by undergoing Richter transformation (RT) [2]. The condition was first described by Maurice Richter in 1928, and the term was used later on to describe a subset of patients with CLL who developed large-cell lymphoma [3]. The 2008 World Health Organization (WHO) classification of hematopoietic tumors defines RT as the transformation of CLL into a more aggressive lymphoma [1]. The most common histological transformation is that into a diffuse large B-cell lymphoma (DLBCL), although transformations into Hodgkin's lymphoma or prolymphocytic leukemia have also been documented [4].

## Review

The risk of RT is independent of disease stage, duration, or response to prior treatment, and it has a poor prognosis with a median survival of less than six months [5]. The clinical features of RT are non-specific: fever, weight loss, night sweats, rapidly enlarging lymph nodes, and suggestive lab findings of elevated lactate dehydrogenase (LDH) levels and beta-2 microglobulin (B2M) levels. However, these features can also be found in patients without the underlying RT [6]. Clinical factors such as advanced Rai stage (3, 4) disease and lymph node size greater than 5 cm are associated with an increased risk of RT [2]. Furthermore, a threefold increase in RT was found in patients who had received a chemotherapy combination of alkylating agents and purine nucleoside analogs [3].

### Molecular diagnostics

Excisional biopsy is the gold standard for diagnosing a patient with RT, but it is not always possible, especially if the patient is symptomatic and immediate treatment is required. At least fine-needle aspiration cytology (FNAC) to confirm the presence of large cells should be performed. Bone marrow biopsy is performed to complete the staging workup. Fluorescence in situ hybridization (FISH) studies help assess the presence of new genetic abnormalities (such as trisomy 12, del 11q, and TP53 mutation) which have prognostic significance in RT.

**Figure 1:**
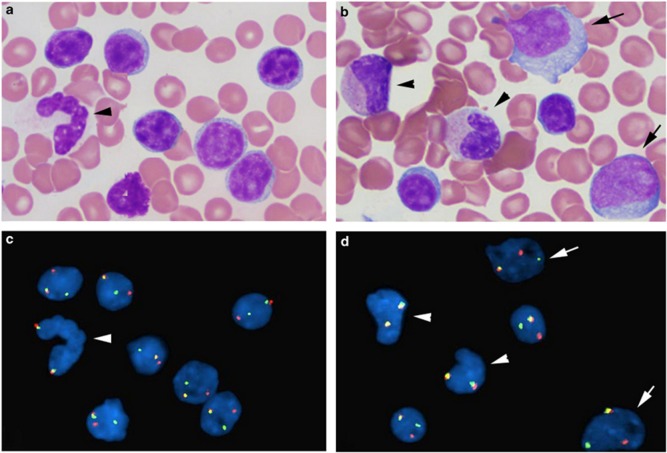
Combined morphologic-FISH analysis shows MYC rearrangement in small mature chronic lymphocytic leukemia cells and large prolymphocytes (a, b): Wright-Giemsa stain of bone marrow aspirate smear; (c, d): FISH analysis with MYC break-apart probe on the same slides as (a) and (b). Arrow head: granulocyte and myelocyte; arrow: prolymphocytes; others are small CLL cells. (Reference: Yan Li, et al. [12]).

RT results in a heterogeneous disorder with some lymphomas evolving from the clonal CLL population and others developing independently [7]. Clonally unrelated cases behave similarly to de-novo DLBCL with similar outcomes and most commonly demonstrate immunoglobulin heavy chain variable region (IgVH) mutation [8]. Conventional cytogenetic analyses and FISH have shown that patients with RT are more likely to have complex cytogenetic abnormalities with no clear recurrent anomalies [9]. MYC translocation is extremely rare in CLL but may play a role in the development of this transformation [3]. RT has a genomic complexity situated between the genomic complexity of CLL and DLBCL [10], and inactivation of tumor protein p53 (TP53) and cyclin-dependent kinase inhibitor 2A (CDKN2A) was found in half the cases [11]. Yan Li, et al. reported that 8q24/MYC rearrangement in chronic lymphocytic leukemia may be associated with RT (see Figure 1 for morphologic-FISH analysis of MYC rearrangement in small mature CLL cells and large prolymphocytes) [12].

CLL patients with stereotype B-cell receptors (BCR) have a higher risk of transformation, independent of IgVH mutation status, which suggests the role of antigen stimulation in RT [3]. Aydin, et al. found that a single nucleotide polymorphism (SNP) leading to C>G variation at position 184 (rs6449182) on the CD38 gene was associated with a higher risk of RT [13-14]. High-risk genomic aberrations (such as del11q and del17p) detected by FISH at the time of CLL diagnosis were associated with an increased risk of future RT as well [15]. Recent studies have shown that patients with mutations in NOTCH1 have a significantly higher probability of RT (30%–45% at 10–15 years) compared to those without them [16]. Multivariate analysis has identified six predictive factors for RT: NOTCH1 mutation, IgVH status, trisomy 12, del 11q22-23, TP53 mutation, and CD38 positivity (30% or more) [17-18].

### FDG PET/CT imaging

**Figure 2:**
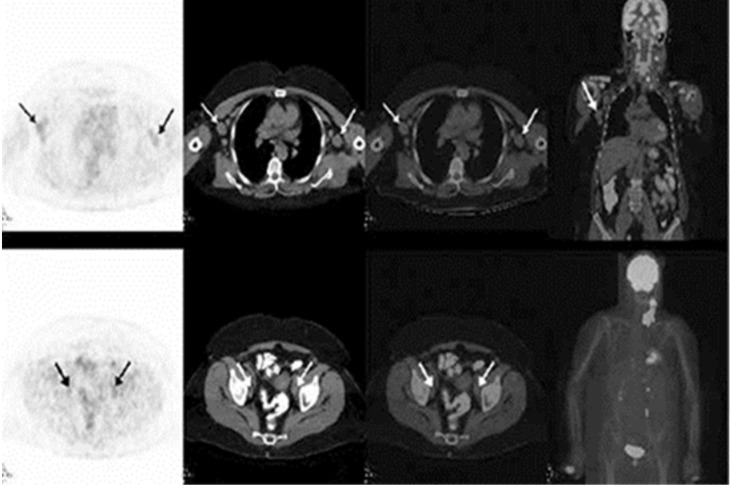
FDG-PET/CT images demonstrating minimally FDG-avid axillary and pelvic lymph nodes suggestive of indolent lymphoma A 64-year-old female with a diagnosis of CLL/SLL. Axial PET and fused axial and coronal PET/CT images demonstrated minimally FDG-avid right cervical, right supraclavicular, and bilateral axillary nodes (upper row), and minimally FDG-avid bilateral internal iliac nodes (lower row), which was compatible with the low FDG uptake of indolent lymphoma. (Reference: Yilmaz, et al. [21]).

**Figure 3:**
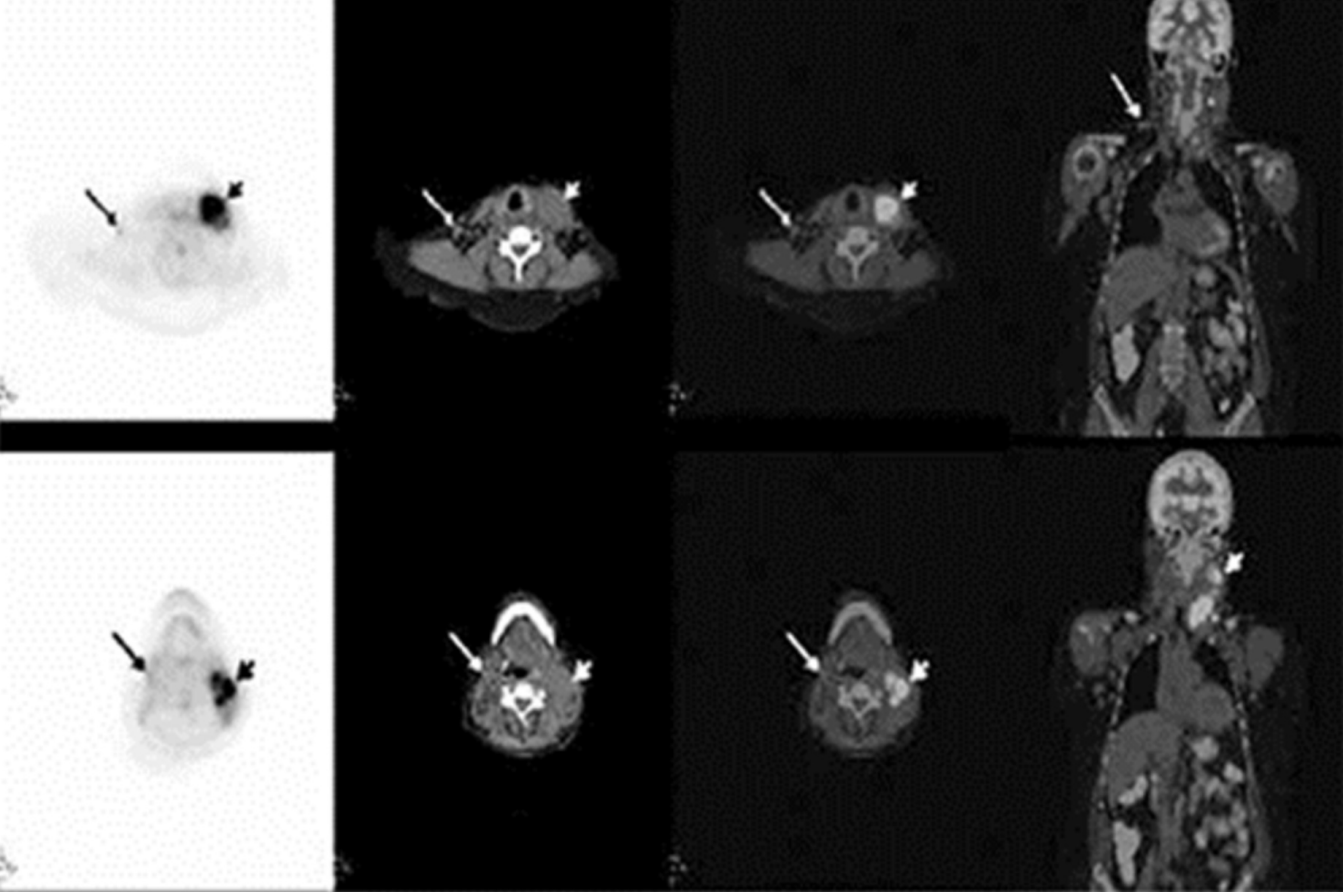
FDG-PET/CT images demonstrating intensely FDG-avid left cervical nodes suggestive of Richter transformation Axial PET, CT, and fused PET/CT images revealed left submandibular and upper-middle jugular conglomerate lymph nodes (short arrow) with intense FDG uptake (SUVmax 13), suggesting transformation. Additionally, minimally increased FDG uptake in the right jugular chain (long arrow) compatible with indolent lymphoma involvement was noted. (Reference: Yilmaz, et al. [21]).

Indolent lymphomas are not considered to be FDG-avid on PET imaging, which is likely due to the lower mitotic activity and glucose consumption of lymphocytes. However, transformation to an aggressive lymphoma enhances their glucose consumption, making the lymphoid tissue highly FDG-avid [19]. Therefore, an interval increase in the standardized uptake value (SUV) in a nodal adenopathy raises the suspicion for RT, especially if above 5 SUVmax, and should be further investigated for histopathologic correlation. Furthermore, FDG-PET/CT serves the additional purpose of guiding the biopsy by identifying intensely metabolically active nodes that are more likely to demonstrate RT. The overall sensitivity and specificity of PET/CT for RT was reported to be 91% and 80%, respectively, with a SUVmax threshold of five [19]. A relatively lower specificity and negative predictive value (80% and 53%, respectively) of FDG-PET for RT was reported, but this could be attributed to the inability of PET to distinguish between RT and other FDG-avid malignancies [19]. Papajik, et al. make the case that there is no advantage in performing FDG-PET/CT over CT as a surveillance tool in patients with CLL, but if RT is suspected given the clinical or CT findings, performing an FDG-PET/CT can be extremely beneficial in confirming it [20]. The following case, reported by Yilmaz, et al., demonstrates the ability of FDG-PET/CT to detect RT in an intensely FDG-avid left cervical lymph node as opposed to minimally FDG-avid bilateral axillary and pelvic nodes that were consistent with indolent lymphomas (see Figures 2-3) [21].

**Figure 4:**
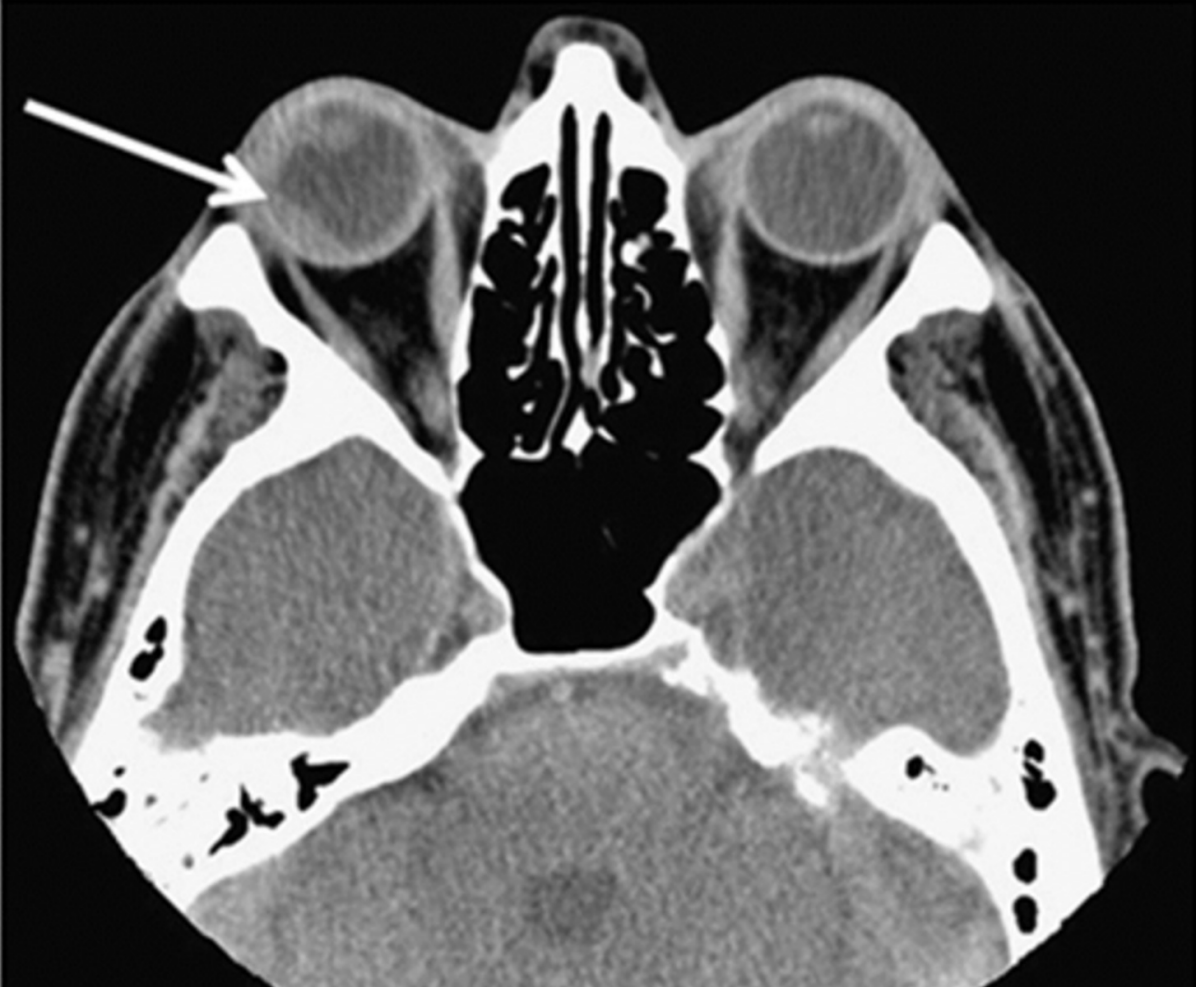
CT image demonstrating right ocular lymphoma lesion with extrascleral involvement CT scan showing a chorioretinal mass (arrow) in the right orbit, predominantly affecting the lateral and posterior parts of the globe, as well as transscleral extension laterally at the time of choroidal biopsy. (Reference: Mota, et al. [22]).

Conventional anatomic imaging such as CT continue to be the standard modality to evaluate lymphomatous lesions with compressive or infiltrative patterns. In the next case reported by Mota, et al., the CT scan clearly demonstrates an ocular lymphoma with RT, including transscleral invasion (see Figure 4) [22].

Another nuclear medicine study, called Gallium-67 scintigraphy, has successfully detected RT in some cases. However, it has fallen out of favor as a desirable scan to detect RT given its limited availability, the need to scan the patient within 24 hours of the injection, and its diminished ability to detect disease in areas of biodistribution (liver, spleen, abdomen, and inguinal region) [23].

### Therapeutic management

Treatment for RT differs from CLL treatment and is associated with greater toxicity. Clinicians must consider prior chemotherapy exposure and the possibility of chemo-refractory disease, which may limit treatment options [3]. Historically, anthracycline-based chemotherapy (CHOP) has been widely used because of its safer toxicity profile. The addition of rituximab (R-CHOP) increases the overall response rates to 39% overall response and 12% complete response [24]. Novel agents such as ibrutinib (Bruton’s tyrosine kinase inhibitor), idelalisib (a phosphatidylinositide 3-kinase inhibitor), and venetoclax (ABT-199/GDC-0199, a BCL-2 antagonist) are used [25]. These agents may have an increased efficacy in treating CLL if RT is detected.

## Conclusions

There are multiple diagnostic studies to stratify CLL and predict RT, including FISH, PCR, and other molecular examinations. FDG-PET/CT imaging can play an important role in its early detection, based on high metabolic activity within nodal stations that are undergoing the RT, as well as in biopsy guidance. Together, these can have a synergistic impact on RT management as well as the potential to improve the survival outcome in patients with this high-grade malignant disease.
